# Reconsidering Tonotopic Maps in the Auditory Cortex and Lemniscal Auditory Thalamus in Mice

**DOI:** 10.3389/fncir.2017.00014

**Published:** 2017-02-28

**Authors:** Hiroaki Tsukano, Masao Horie, Shinpei Ohga, Kuniyuki Takahashi, Yamato Kubota, Ryuichi Hishida, Hirohide Takebayashi, Katsuei Shibuki

**Affiliations:** ^1^Department of Neurophysiology, Brain Research Institute, Niigata UniversityNiigata, Japan; ^2^Division of Neurobiology and Anatomy, Graduate School of Medicine and Dental Sciences, Niigata UniversityNiigata, Japan; ^3^Division of Otolaryngology, Graduate School of Medicine and Dental Sciences, Niigata UniversityNiigata, Japan

**Keywords:** brain map, auditory cortex, medial geniculate body, tonotopy, topology, thalamocortical pathway, multiple compartments, mice

## Abstract

The auditory thalamus and auditory cortex (AC) are pivotal structures in the central auditory system. However, the thalamocortical mechanisms of processing sounds are largely unknown. Investigation of this process benefits greatly from the use of mice because the mouse is a powerful animal model in which various experimental techniques, especially genetic tools, can be applied. However, the use of mice has been limited in auditory research, and thus even basic anatomical knowledge of the mouse central auditory system has not been sufficiently collected. Recently, optical imaging combined with morphological analyses has enabled the elucidation of detailed anatomical properties of the mouse auditory system. These techniques have uncovered fine AC maps with multiple frequency-organized regions, each of which receives point-to-point thalamocortical projections from different origins inside the lemniscal auditory thalamus, the ventral division of the medial geniculate body (MGv). This precise anatomy now provides a platform for physiological research. In this mini review article, we summarize these recent achievements that will facilitate physiological investigations in the mouse auditory system.

## Introduction

The auditory ascending pathway is an important system that conveys sound information to the auditory cortex (AC) in mammals. The pathway that originates from the ear is called the lemniscal pathway, which passes, among others, through the central nucleus (ICc) of the inferior colliculus (IC) in the midbrain, and the ventral division (MGv) of the medial geniculate body (MGB) in the thalamus, en route to the AC (Lee and Sherman, 2010; Lee et al., 2011; Winer and Schreiner, 2011). To understand mammalian audition, it is crucial to reveal one by one the functional roles of these auditory nuclei and the subregions of the AC. For example, a distinct function of the IC has been revealed: detecting sound localization by reference to interaural time differences (Fujita and Konishi, 1991; Grothe et al., 2010). This success in the IC was achieved by studies using an appropriate animal model, a barn owl, which has outstanding ability for sound localization. Yet, little is known about the auditory thalamus and AC, probably because they have very complex functions and working mechanisms. To investigate the MGv and AC, a suitable animal model is needed in which various experimental tools are available to observe the many-sided aspects of the central auditory system.

In the last 10 years, the mouse has emerged as an animal model that is amenable to auditory research. Mice have been used for physiological analyses of tonotopy (Bandyopadhyay et al., 2010; Rothschild et al., 2010; Guo et al., 2012; Winkowski and Kanold, 2013; Issa et al., 2014; Barnstedt et al., 2015), development (Barkat et al., 2011), reward-related plasticity (Ohshima et al., 2010; Kato et al., 2015), relationships with hormones (Marlin et al., 2015) and behavior (Schneider et al., 2014), multimodal interactions (Lesicko et al., 2016), and aging (Brewton et al., 2016). However, the use of mice in central auditory system research is still limited despite its advantages, which include sophisticated genetic tractability (Yang et al., 2013). Research involving other regions of the cortex, especially the visual cortex (VC), has delineated ever finer cortical surface maps in mice (Garrett et al., 2014), which, in turn, has revealed distinct regional functional properties (Juavinett and Callaway, 2015) and connectivity (D’Souza et al., 2016). Accordingly, the mouse has become an essential platform for vision research. Therefore, delineating an anatomically precise figure of the mouse auditory system is essential to enable further physiological research regarding the function of the central auditory system. In this mini review article, we briefly describe the macroscopic auditory thalamocortical structures that have so far been uncovered in the mouse.

## Multiple Tonotopic Regions in the Mouse Auditory Cortex

Neuroscience studies are today performed according to the theory of functional specialization; the mammalian brain is divided into functional modules by location (Kanwisher, 2010; Zilles and Amunts, 2010). Considering this principle, the AC is further divided into several subregions, each of which should have a distinct regional function for sound processing. The spatial arrangement of these subregions is generally represented and illustrated as an auditory cortical map. Thus, a more detailed AC map provides a better platform for investigating distinct regional function because all physiological investigations are conducted on the basis of this map.

**Figure 1:**
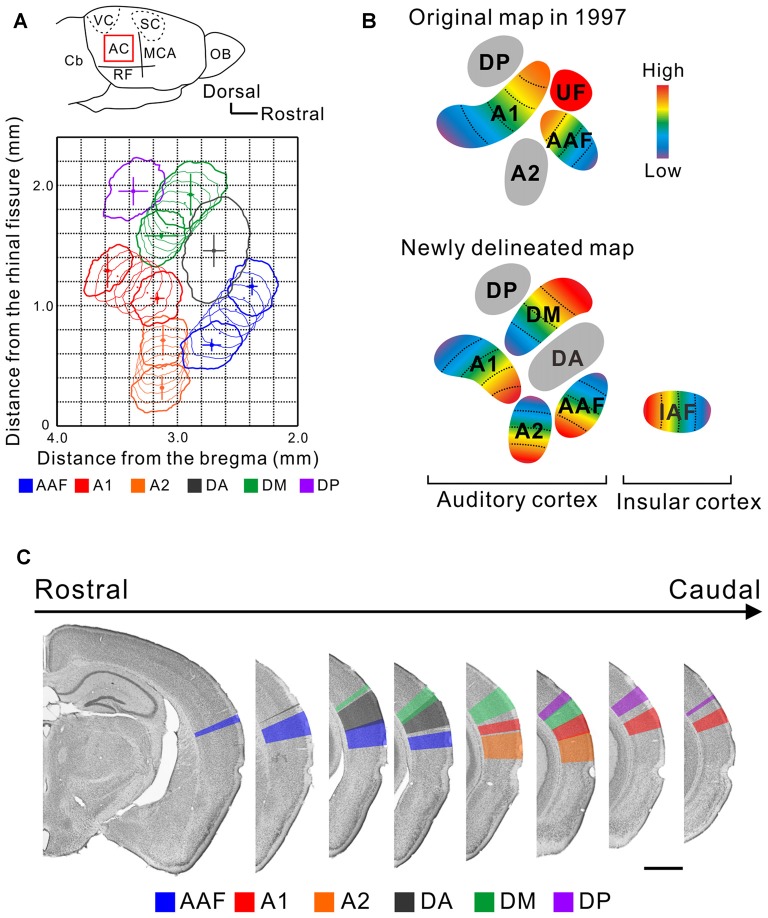
**A new map of the mouse auditory cortex visualized using flavoprotein fluorescence imaging (FFI). (A)** A quantitative surface map of the six subregions of the mouse AC revealed using FFI. These panels were modified from Tsukano et al. (2016). AC, auditory cortex; Cb, cerebellum; MCA, medial cerebral artery; OB, olfactory bulb; RF, rhinal fissure; SC, somatosensory cortex; VC, visual cortex. **(B)** Illustration of the original map (Stiebler et al., 1997) and a map based on the results of recent optical imaging studies (Sawatari et al., 2011; Tsukano et al., 2015). These AC maps are considered to reflect a map lying in layers 2/3. **(C)** Delineation of the six auditory subregions in the coronal view. Bar, 1 mm. These panels were modified from Tsukano et al. (2016).

The mouse AC map was first described about two decades ago (Stiebler et al., 1997). This achievement is praiseworthy because the researchers identified multiple auditory regions without any prior knowledge by investigating the distribution of a characteristic frequency (CF), a frequency for which a neuron has its lowest excitatory threshold, using unit recording. This map represented the AC with five subregions; two tonotopic regions, the anterior auditory field (AAF) and primary auditory cortex (A1), and three non-tonotopic regions, the secondary auditory field (A2), ultrasonic field (UF), and dorsoposterior field (DP; Figure 1). Remarkably, “UF” was set as a region in the dorsorostral corner of the AC where neurons with a CF over 40 kHz were localized while the tonotopy of the AAF and A1 was limited to less than about 40 kHz. The presence of the segregated “UF” might be regarded as a feature or symbol of the AC in mice which use ultrasonic sounds over 40 kHz in vocal communication (Ehret, 1987; Holy and Guo, 2005; Asaba et al., 2014).

Optical imaging which enables uniform observation of the brain surface (Hishida et al., 2014; Matsui et al., 2016) will likely become an additional or alternative tool to visualize the fine mouse AC maps. Here, we discuss the advantages and disadvantages of mapping using optical imaging vs. electrophysiology. Optical imaging does not require invasive operations, such as dense penetration of electrodes into the brain, as it allows the visualization of neural responses on the cortical surface at a glance. Of note, flavoprotein fluorescence imaging (FFI; Shibuki et al., 2003) and imaging using Cre-dependent GCaMP-expressing mice (Zariwala et al., 2012), both of which observe neural responses via originally- and homogenously-expressed fluorophores in the brain, require neither a craniotomy nor calcium-sensitive-dye application, thus permitting transcranial observation. Indeed, these methods have been used to visualize fine responses on the cortical surface of the primary (Yoshitake et al., 2013) and higher-order visual areas (Tohmi et al., 2009, 2014; Andermann et al., 2011). However, optical imaging has several disadvantages compared with electrophysiology. First, with the exception of voltage-sensitive-dye imaging, optical imaging has a poor temporal resolution. Second, it is unclear from which layers signals are detected. It is assumed that optical imaging visualizes responses in layers 2/3 in mice: this is because physiological properties observed using optical imaging are consistent with those of layer 2/3 neurons observed using two-photon imaging (Tohmi et al., 2014), and because the permeability of blue excitation light is relatively low. Third, electrophysiology is useful for investigating deep brain regions such as the thalamus (Hackett et al., 2011). Finally, optical imaging is unavailable for single-neuron scale analyses. After understanding the merits and demerits, it is clear that the selection of an appropriate technique is dependent on the purpose of the investigations.

Taking advantage of abovementioned merits, optical imaging has enabled the visualization of fine responses in small auditory regions, permitting us to propose a new map of the mouse AC. Mouse AC maps generated using FFI and imaging involving GCaMP3-expressing mice are different from classical maps in the following ways: (1) The size and location of the auditory regions are symmetrical in both hemispheres, at least in C57BL/6 mice (Tsukano et al., 2016) although the left AC has traditionally been considered to be larger than the right (Stiebler et al., 1997). (2) The region that was classically annotated as A1 is divided into two tonotopic regions, A1 and the dorsomedial field (DM; Tsukano et al., 2013a, 2015, 2017). Actually, dense high-quality electrophysiological mapping succeeded in distinguishing these two regions (Guo et al., 2012), as shown in Figure 7 in Issa et al. (2014). (3) The A2 has a tonotopic arrangement that runs dorsoventrally (Kubota et al., 2008; Issa et al., 2014; Tsukano et al., 2015, 2016). (4) The tonotopic direction of the AAF travels dorsoventrally (Tsukano et al., 2015, 2016; Issa et al., 2014). (5) Overall, at least six subregions exist in the mouse AC (Figure 1). Four regions, the AAF, A1, A2, and DM, are tonotopically arranged. Two regions, the dorsoanterior field (DA) and DP, are non-tonotopic regions. Single-neuron scale analyses showed that neurons in these non-tonotopic regions have a distinct CF (Guo et al., 2012) but their spatial distribution is randomized (Stiebler et al., 1997; Honma et al., 2013). The new auditory cortical map is supported by anatomical investigations that show regional differences in cytoarchitectural patterns visualized by immunolabeling of non-phosphorylated neurofilaments (NNF). Auditory regions have distinct NNF staining patterns in terms of dendritic arborization and distribution by layer in mice (Horie et al., 2015), as shown in another rodent study (Budinger et al., 2000). Moreover, auditory regions have distinct thalamic origins (Horie et al., 2013; Takemoto et al., 2014; Tsukano et al., 2015). (6) The last point is the most important to accentuate; the independent UF is unlikely to be present in the mouse AC. This claim is supported by the fact that all four of the tonotopic regions (the AAF, A1, A2 and DM) include distinct ultrasonic frequency bands over 40 kHz (Figure 1; Issa et al., 2014; Tsukano et al., 2015, 2016). Therefore, the term UF can be considered obsolete. We assume that the region that was first annotated as the UF was a mixture of the DA and high frequency bands of the DM. A key sentence in Stiebler et al. (1997) supports this idea: “Best frequencies^1^ of neurons in the UF were often difficult to determine because—especially in its rostral part—neurons preferentially responded to frequency-modulated tones”. (p. 561, line 14 from the bottom). Optical imaging also indicated that there is a non-tonotopic region, the DA, that responds well to frequency modulation sounds near the UF (Honma et al., 2013; Tsukano et al., 2015). Although dense electrode mapping does not clearly support the presence of the DA (Guo et al., 2012), further investigations will be likely to resolve this discrepancy by surveying another parameter as electrophysiology has the advantage of investigating single-neuron level properties (Joachimsthaler et al., 2014). Overall, the presence of the UF region has been a major obstacle when comparing the mouse AC with those of other rodents because a UF-like region is absent even in the rat, a rodent very similar to the mouse. By abandoning the UF we now have the possibility to homologize or analogize the auditory cortices of different rodents, facilitating physiological research based on cortical spatial information (Baba et al., 2016).

## Multiple Compartments in the Mouse MGv and Parallel Processing in the Central Lemniscal Auditory System

It is well known that tonotopy originates in the cochlea. Sounds enter the ears and the vibrations are transmitted to the basilar membrane in the cochlea. Frequencies of tones are converted into a one-dimensional spatial arrangement on the basilar membrane and arrayed as a single gradation from low to high frequencies (Békésy, 1960). The tonotopic gradient is conveyed through the central auditory ascending pathway, the ICc and MGv, en route to the A1 (Lee and Sherman, 2010; Lee et al., 2011), where nuclei are connected topologically. Therefore, there is a prevailing concept that only a single tonotopic gradient exists in the ICc (Portfors et al., 2011; Cheung et al., 2012) and MGv (Cetas et al., 2001; Hackett et al., 2011; Moerel et al., 2015) across species, although it may diverge or be duplicated when entering the AC.

**Figure 2:**
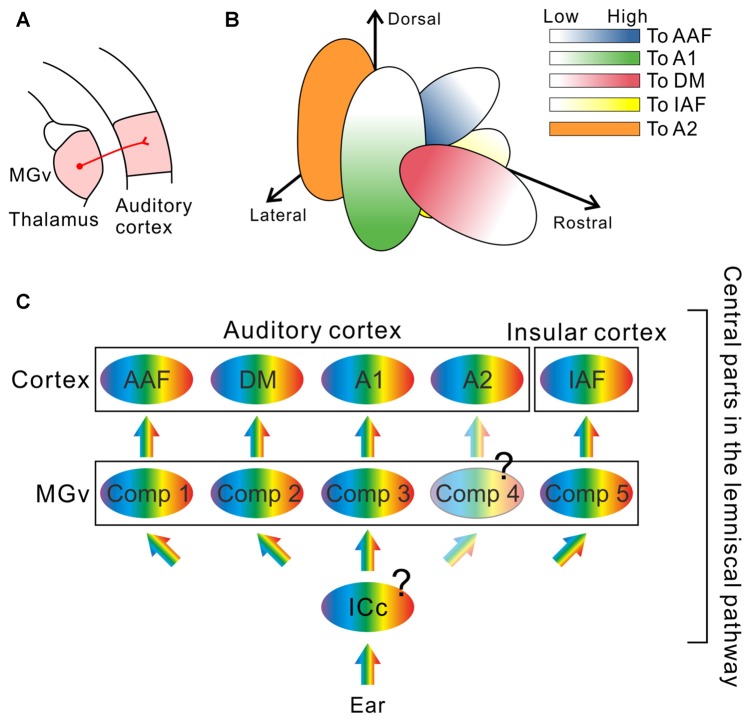
**The remodeled thalamocortical auditory pathway in mice. (A)** Schematic drawing of the thalamocortical auditory pathway. The AC receives thalamic inputs from the ventral division of the medial geniculate body (MGv). **(B)** Multiple compartments revealed inside of the MGv. Neurons projecting to the dorsomedial field (DM) are localized in the rostral compartment (red; Tsukano et al., 2015). Neurons projecting to the primary auditory cortex (A1) are localized in the lateral compartment of the middle MGv (green; Horie et al., 2013; Takemoto et al., 2014). Neurons projecting to the anterior auditory field (AAF) are localized in the medial compartment of the middle MGv (blue; Horie et al., 2013; Takemoto et al., 2014). Neurons projecting to the insular auditory field (IAF) are localized in the inferomedial compartment in the middle MGv (yellow; Takemoto et al., 2014). Each compartment gives rise to topological projections to its cortical target. In addition, the caudal half of the mouse MGv is now uncharacterized (Tsukano et al., 2015); therefore, it is highly possible that neurons projecting to the secondary auditory field (A2), a remaining tonotopic region in the AC, are localized in the caudal MGv compartment as suggested in Ohga et al. (2015). **(C)** A new model of the thalamocortical auditory pathway, which is composed of several parallel streams. Future studies are necessary to determine whether the central nucleus of the inferior colliculus (ICc) is also composed of multiple compartments with a distinct frequency organization, or whether divergence of tonotopy from the ICc to the MGv occurs. Moreover, whether that the caudal compartment in the MGv is arranged topographically is not clear. Comp, compartment.

However, recent investigations revealed new structures in the auditory thalamus that may be involved in essential auditory processing: The mouse MGv is not arranged as a single monotonic structure but is composed of multiple compartments, each of which gives rise to frequency-related topological projections to distinct cortical targets (Figure 2). Horie et al. (2013) and Takemoto et al. (2014) injected retrograde tracers along tonotopic axes in auditory cortical regions identified using optical imaging, and found compartments projecting to the AAF, A1, or insular auditory field (IAF) in the middle of the MGv. Although previous studies using cats suggested the presence of parallel projections in the lemniscal thalamocortical pathways (Huang and Winer, 2000; Lee et al., 2004; Lee and Winer, 2008), clear multiple compartments with distinct tonotopy in the MGv were not reported. Here, we must note that topography in the mouse MGv cannot currently be equated with tonotopy. While tonotopic gradients in the AC have been studied in detail, those in the MGv have not, and few studies have investigated whether identical frequency bands in the MGv and AC are topologically connected (although one study in cats has confirmed this (Lee et al., 2004)). These results were recently challenged in mice, using combination of tracing and electrophysiology, to confirm that two tonotopic gradients in the MGv and AC are connected via topological projections (Hackett et al., 2011). The authors clearly showed that tonotopy in at least one cortical region and that in the corresponding MGv compartment are connected in a topological fashion. In addition, their data showed CF distribution in a middle-middle-high-low fashion in the middle MGv in the lateromedial axis, which is consistent with the arrangement suggested by tracing experiments (Horie et al., 2013; Takemoto et al., 2014), although this study was conducted with the assumption that tonotopy of the MGv would be monotonic. Now that the rostral compartment has been found to project to the DM (Tsukano et al., 2015), at least three parallel topological connections between the MGv and AC and one topological connection between the MGv and IAF have been revealed in mice (Figure 2). Therefore, future physiological studies are necessary to confirm that all topographic organization in the MGv, which is related to cortical frequency gradients, is consistent with the “low to high” gradient of CF distribution of MGv neurons.

The presence of such macroscopic structure-based parallel pathways in the lemniscal pathway strongly indicates that cortical multiple tonotopy could be established by multiple topological thalamocortical inputs (Figure 2). Note that the corticocortical formation and thalamocortical formation of tonotopy in the cortex are not mutually exclusive. In the prevailing concept that auditory information is conveyed through the intracortical hierarchical stream starting from A1, multiple tonotopic organization is considered to reflect A1 tonotopy: this is undoubtedly because isofrequency bands in cortical tonotopy are connected with one another (Schreiner and Winer, 2007; Lee and Winer, 2008). In contrast, MGv compartments send topological projections towards layers 3b/4 in auditory cortical subregions. There, thalamocortical and corticocortical inputs may converge on dendrite trees of thalamorecipient neurons (Richardson et al., 2009). Tonotopic organization in layers 3b/4 is further conveyed to layer 2/3, largely keeping the original form (Guo et al., 2012), although the micro-scale complexity increases (Bandyopadhyay et al., 2010; Rothschild et al., 2010; Winkowski and Kanold, 2013). Overall, auditory cortical maps observed via optical imaging are established as a result of integration of the thalamocortical and corticocortical formation.

Importantly, the presence of lemniscal parallel pathways suggest the possibility that distinct auditory information is conveyed to cortical regions other than A1 directly from the MGv. In the prevailing concept, the auditory information first enters the core regions and is then relayed to higher order auditory fields (Kaas and Hackett, 2000). However, even the DA, which is considered to be a higher-order region because it lacks tonotopy, receives dense projections directly from the MGv (Hofstetter and Ehret, 1992; Honma et al., 2013; Tsukano et al., 2013b, 2015) although the precise locations of the projection neurons have not yet been quantitatively analyzed. The MGv must have a gating function to decide what sound information is to be sent to each cortical target. It is well known that the sensory thalamus has close relationships with the thalamic reticular nucleus (TRN), which is occupied with GABAergic neurons and is involved in the gate control of ascending auditory inputs (Cotillon-Williams et al., 2008; Kimura et al., 2009). It has become more likely that the MGv acts not only as a relay point but as a selection filter of auditory information (Blundon and Zakharenko, 2013).

A macroscopic structure-based thalamocortical parallel pathway may be ubiquitous in rodent sensory systems. It has been reported that the auditory system in rats has parallel thalamocortical pathways. The rat AC includes multiple tonotopic regions (Kalatsky et al., 2005; Polley et al., 2007), similar to the mouse, and the thalamic origins of these tonotopic regions: A1 and the ventral part of the AC, are rostrocaudally different in the MGv (Storace et al., 2011, 2012; Shiramatsu et al., 2016). Thus, spatial relationships among thalamic origins and cortical targets are similar between mice and rats. Moreover, older tracing experiments hinted at the existence of parallel auditory thalamocortical pathways in another rodent species, the guinea pig (Redies et al., 1989). In the mouse VC, detailed higher-order regions have been revealed using optical imaging (Tohmi et al., 2009, 2014; Andermann et al., 2011; Marshel et al., 2011) and their thalamic origins from the visual thalamus were different, suggesting the presence of parallel streams in the higher-order visual system (Tohmi et al., 2014). Future physiological studies should consider which aspects of sensory information are fed into cortical subregions through the thalamus, and examine how parallel thalamic afferents cooperate with corticocortical hierarchical processing.

The origin of lemniscal tectothalamic projections to the MGv is the ICc. As mentioned above, the ICc is considered to be a monotonic, single structure (Figure 2). However, the ICc may also be composed of multiple compartments with distinct tonotopy, each of which sends projections to a compartment in the MGv. Otherwise, tonotopy in the ICc may be single but tonotopy diverges when transmitted to the MGv such that a single neuron gives rise to projectional branches towards several compartments in the MGv. It is important to know in which lemniscal nucleus tonotopic divergence occurs in terms of gating or selection of sound features by pathway. At least, we need to admit that audition is realized by more complex pathways than previously thought.

## Conclusions

A detailed map of the AC and new scheme of parallel thalamocortical projections from the MGv and AC have been gradually revealed in mice, leading to the concept that cortical multiple tonotopy represents “multiple cores” in rodents (Storace et al., 2012). A new theory or model will be necessary to combine the multiple parallel peripheral inputs into the multiple cortical regions with existing mammalian corticocortical hierarchical processing (Felleman and Van Essen, 1991; Kaas and Hackett, 2000). Because the functional significance of the presence of tonotopy is controversial today (Hackett et al., 2011; Aschauer and Rumpel, 2014), further studies are necessary to determine why both the auditory thalamus and cortex require multiple compartments and regions with distinct tonotopy. Based on the theory of functional specialization, each auditory cortical subregion and compartment may have a distinct role to process a distinct sound factor. These questions are essential for future central auditory system research to reveal working mechanisms.

## Author Contributions

HTsukano, MH, SO, KT and YK conducted experiments and obtained basic knowledge to write this review. RH and HTakebayashi provided critical idea and comments to this work. HTsukano obtained funding for this work. HTsukano wrote the manuscript, and HTsukano and KS revised it. All the authors approved the publication of this manuscript.

## Funding

This work was supported by Japan Society for the Promotion of Science (JSPS) KAKENHI Grant No. 26830008 (to HTsukano), a grant for the Promotion of Medical Science and Medical Care No. 15KI149 from the Ichiro Kanehara Foundation (to HTsukano), and a grant for Basic Science Research Projects No. 140254 from the Sumitomo Foundation (to HTsukano).

## Conflict of Interest Statement

The authors declare that the research was conducted in the absence of any commercial or financial relationships that could be construed as a potential conflict of interest.

## Footnotes

^1^Today, the best frequency (BF) in the auditory system is usually defined as a frequency to which a neuron is the most sensitive under the condition that tonal intensity is constant. Although the term BF was used in their article, it can be considered as a characteristic frequency (CF) precisely.

## Abbreviations

AAF: anterior auditory field
AC: auditory cortex
A1: primary auditory field
A2: secondary auditory field
CF: characteristic frequency
DA: dorsoanterior field
DM: dorsomedial field
DP: dorsoposterior field
FFI: flavoprotein fluorescence imaging
IAF: insular auditory field
IC: inferior colliculus
ICc: central nucleus of the inferior colliculus
MGB: medial geniculate body
MGv: ventral division of the medial geniculate body
TRN: thalamic reticular nucleus
UF: ultrasonic field.
