# Corrigendum: Reconsidering Tonotopic Maps in the Auditory Cortex and Lemniscal Auditory Thalamus in Mice

**DOI:** 10.3389/fncir.2017.00039

**Published:** 2017-05-31

**Authors:** Hiroaki Tsukano, Masao Horie, Shinpei Ohga, Kuniyuki Takahashi, Yamato Kubota, Ryuichi Hishida, Hirohide Takebayashi, Katsuei Shibuki

**Affiliations:** ^1^Department of Neurophysiology, Brain Research Institute, Niigata UniversityNiigata, Japan; ^2^Division of Neurobiology and Anatomy, Graduate School of Medicine and Dental Sciences, Niigata UniversityNiigata, Japan; ^3^Division of Otolaryngology, Graduate School of Medicine and Dental Sciences, Niigata UniversityNiigata, Japan

**Keywords:** brain map, auditory cortex, medial geniculate body, tonotopy, topology, thalamocortical pathway, multiple compartments, mice

We noticed that there was an error in the illustration in **Figure 2B**. In particular, we inadvertently drew the red oval in the opposite direction to the original data in the references (Tsukano et al., 2015, 2017). There are no relevant errors in the text part.

**Figure 1:**
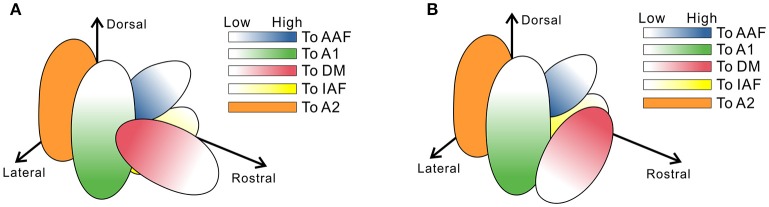
**(A)** The original illustration in **Figure 2B**. **(B)** A corrected illustration to **Figure 2B**. The topography of the rostral compartment in the ventral division of the medial geniculate body (MGv), shown in red, travels largely from ventrorostrolateral to dorsocaudomedial according to Tsukano et al. (2015, 2017).

## Conflict of interest statement

The authors declare that the research was conducted in the absence of any commercial or financial relationships that could be construed as a potential conflict of interest.

## References

[B1] Tsukano H. Horie M. Bo T. Uchimura A. Hishida R. Kudoh M.. (2015). Delineation of a frequency-organized region isolated from the mouse primary auditory cortex. J. Neurophysiol. 113, 2900–2920. 10.1152/jn.00932.2014 25695649 PMC4416634

[B2] Tsukano H. Horie M. Hishida R. Takahashi K. Takebayashi H. Shibuki K. (2017). Independent tonotopy and thalamocortical projection patterns in two adjacent parts of the classical primary auditory cortex in mice. Neurosci. Lett. 637, 26–30. 10.1016/j.neulet.2016.11.062 27914952

